# Phrenic nerve stimulation for the treatment of central sleep apnea in patients with heart failure

**DOI:** 10.1007/s11325-022-02699-8

**Published:** 2022-08-17

**Authors:** Youmeng Wang, Juliane Schoebel, Jinming Han, Jan F. Kraemer, Theresa Toncar, Jacob Siegert, Thomas Penzel, Christoph Schöbel

**Affiliations:** 1 Sleep Medicine Center, Charité-Universitätsmedizin, Charitéplatz 1, 10117 Berlin, Germany; 2 Department of Neurology, Xuanwu Hospital, Capital Medical University, Beijing, 100053 China; 3 Department of Physics, Humboldt Universität Zu Berlin, Berlin, Germany; 4 Universitätsmedizin Essen, Ruhrlandklinik-Westdeutsches Lungenzentrum, am Universitätsklinikum Essen GmbH, Tüschener Weg 40, 45239 Essen, Germany

**Keywords:** Heart failure, Central sleep apnea, Phrenic nerve stimulation

## Abstract

**Objective:**

Central sleep apnea (CSA) is associated with increased morbidity and mortality in patients with heart failure (HF). We aimed to explore the effectiveness of phrenic nerve stimulation (PNS) on CSA in patients with HF.

**Methods:**

This was a prospective and non-randomized study. The stimulation lead was inserted into the right brachiocephalic vein and attached to a proprietary neurostimulator. Monitoring was conducted during the implantation process, and all individuals underwent two-night polysomnography.

**Results:**

A total of nine subjects with HF and CSA were enrolled in our center. There was a significant decrease in the apnea–hypopnea index (41 ± 18 vs 29 ± 25, *p* = 0.02) and an increase in mean arterial oxygen saturation (SaO2) (93% ± 1% vs 95% ± 2%, *p* = 0.03) after PNS treatment. We did not observe any significant differences of oxygen desaturation index (ODI) and SaO2 < 90% (T90) following PNS. Unilateral phrenic nerve stimulation might also categorically improve the severity of sleep apnea.

**Conclusion:**

In our non-randomized study, PNS may serve as a therapeutic approach for CSA in patients with HF.

## Introduction

Central sleep apnea (CSA) is common in subjects with heart failure (HF), affecting almost half of subjects with systolic HF and 18 to 30% of subjects with diastolic HF [1–5]. CSA is mainly caused by increased respiratory response to variations in PaCO_2_. This oscillation is caused by heightened respiratory instability. Hyperventilation, circulatory delay, and enhanced cerebrovascular reactivity are three elements that determine respiratory instability in patients with HF [6]. CSA can lead to hypoxia, consequences of increases in arrhythmias and sympathetic drive [7, 8]. In subjects with HF, it has been shown to be an important risk factor for mortality [9].

Phrenic nerve stimulation (PNS) is a new method of treating CSA in HF patients by preserving the physiological breathing pattern during central apnea episodes [10, 11]. Using an implantable device therapy is easier than mask-based positive pressure therapies for patients with HF, then improving therapeutic adherence. The PNS treatment has been supported to be an effective treatment in a previous randomized controlled trial involving 151 patients [12]. Although this new device has been used in a few medical centers around the world, it seems to be a safe and effective approach for treating CSA. Ponikowski et al. [13]. conducted a prospective, non-randomized trial to determine the feasibility of PNS for the treatment of CSA in patients with HF. Thirty-one patients from six centers were selected; 16 of them were able to undergo two nights of polysomnography (PSG). They found PNS could result in significant improvement in the AHI and central apnea index(CAI), and PNS can significantly decrease the incidence of CSA and bring back a more natural breathing pattern in patients with HF. Zhang X et al. [14] showed a significant reduction in AHI and CAI at 6-month follow-up.

Previous studies [12–14] have demonstrated that this treatment is safe, that it can significantly reduce episodes of CSA, and that it provides improvements in crucial polysomnographic indicators. However, studies on the effectiveness of applied PNS in HF patients with CSA are still scarce; therefore, our study provides strong evidence for the effectiveness of PNS.

## Methods

### Participants and data collection

The Remedē System Pivotal Trial is a short-term, prospective, single-center, open-label trial involving patients with CSA. Patients who had a diagnosis of sleep apnea and/or previous polysomnographic (PSG) tests supporting periodic breathing with CSA within the preceding 6 months were eligible for this short-term trial. The participants were then subjected to two more full nights of PSG by study design. Subjects were enrolled if they had an apnea–hypopnea index (AHI) ≥ 15. Patients who had supplementary oxygen, phrenic nerve palsy, severe COPD, unstable angina within 3 months of the study, or poor phrenic nerve capture during neurostimulation were excluded in this study.

### Procedure description

**Fig. 1:**
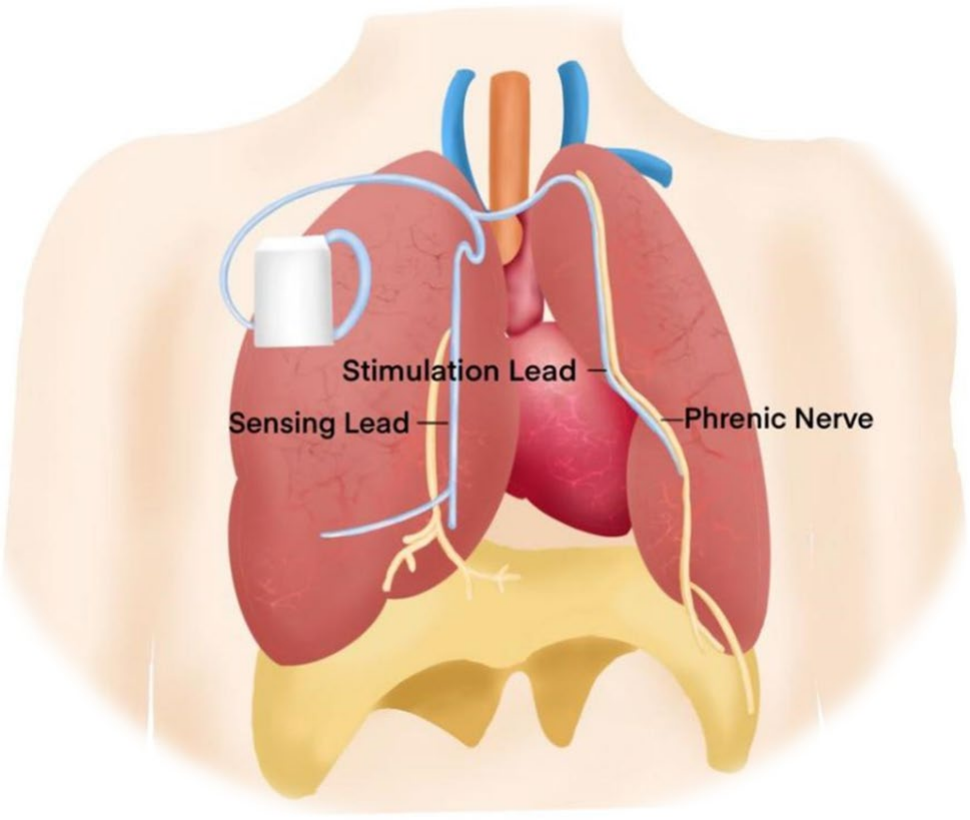
Diagram of phrenic nerve stimulation (Remedē System) treatment

The axillary or subclavian veins were used to gain venous access. To activate the nearby phrenic nerve, stimulation leads (Cardima catheter, USA) were placed in the right brachiocephalic vein **(**Fig. 1**)**. Low-energy nerve stimulation was delivered by an external pulse generator device (Respicardia, Inc.). Capture was determined during the lead implantation operation by external palpation of diaphragmatic contraction on the stimulation side. The level of phrenic nerve stimulation is adjusted as required throughout the evening session, aiming to eliminate centrally mediated apnea episodes that do not disturb the subject.

### Scoring of polysomnographic studies

Two qualified sleep technicians evaluated the two-night PSG. Subject identities, study night ordering and stimulating application were blinded from the technicians. An episode of apnea was characterized as a deficiency of inspiratory airflow over 10 s. Obstructive apnea (OA) was defined as a lack of airflow in the presence of rib and abdominal excursions. Central apnea (CA) was defined as a lack of airflow in the absence of rib and abdominal excursions, as well as a lack of airflow. Hypopnea was defined as a drop in airflow that lasted 10 s or longer accompanied by a drop of at least 4% in arterial oxyhemoglobin saturation.

### Statistical analysis

Descriptive statistics are expressed as standard deviation or numbers and percentages. Paired *t* tests (for data with a normal distribution) and Wilcoxon tests (for data with an abnormal distribution) were performed before and after treatment. Results were considered statistically significant at *p* < 0.05. Data were analyzed using SPSS version 25.0 (New York, USA).

## Results

The characteristics of these nine subjects were summarized in Table 1. Subjects in this study were all male (aged 74.4 ± 8.4 years with a body mass index (BMI) of 28.7 ± 3.5 kg/m^2^). They received standard treatment for HF, and their mean left ventricular ejection fraction (LVEF) was 43 ± 14%. The stimulation lead was positioned in the right brachiocephalic vein for all patients. Three individuals had previously installed cardiac devices. Three patients had a device for cardiac resynchronization treatment (CRT). In the context of PNS, devices were examined for probable over- or under-sensing. To evaluate potential disturbances, the implanted device was programmed with the greatest sensitivity level.

**Table 1:** Baseline characteristics of patients with CHF and CSR

HF patients with CSA (*n* = 9)	
Age (years)	74.4 ± 8.4
Male (%)	100
BMI (kg/m^2^)	28.7 ± 3.5
NYHA class III (%)	100
LVEF (%)	43 ± 14
SBP (mmHg)	117 ± 12
Hemoglobin (g/dL)	14 ± 2
Creatinine (mg/dL)	1.5 ± 0.6
CSA (%)	100
Cardiac infarction (%)	100
Diabetes (%)	33
Hypertension (%)	56
Hyperlipidemia (%)	78
Coronary disease (%)	44
Digitoxin (%)	22
Pandoprazole	44
ARNI	56
ACE/AT1/ARBs	67
CRT (%)	33

*ARNI* angiotensin receptor neprilysin inhibitor, *ACE* angiotensin-converting enzyme, *AT1* angiotensin II Type 1, *ARBs* angiotensin II receptor blockers, *BMI* body mass index, *CSA* central sleep apnea, *CRT* cardiac resynchronization therapy, *NYHA* New York Heart Association, *LVEF* left ventricular ejection fraction, *SBP* systolic blood pressure

**Fig. 2:**
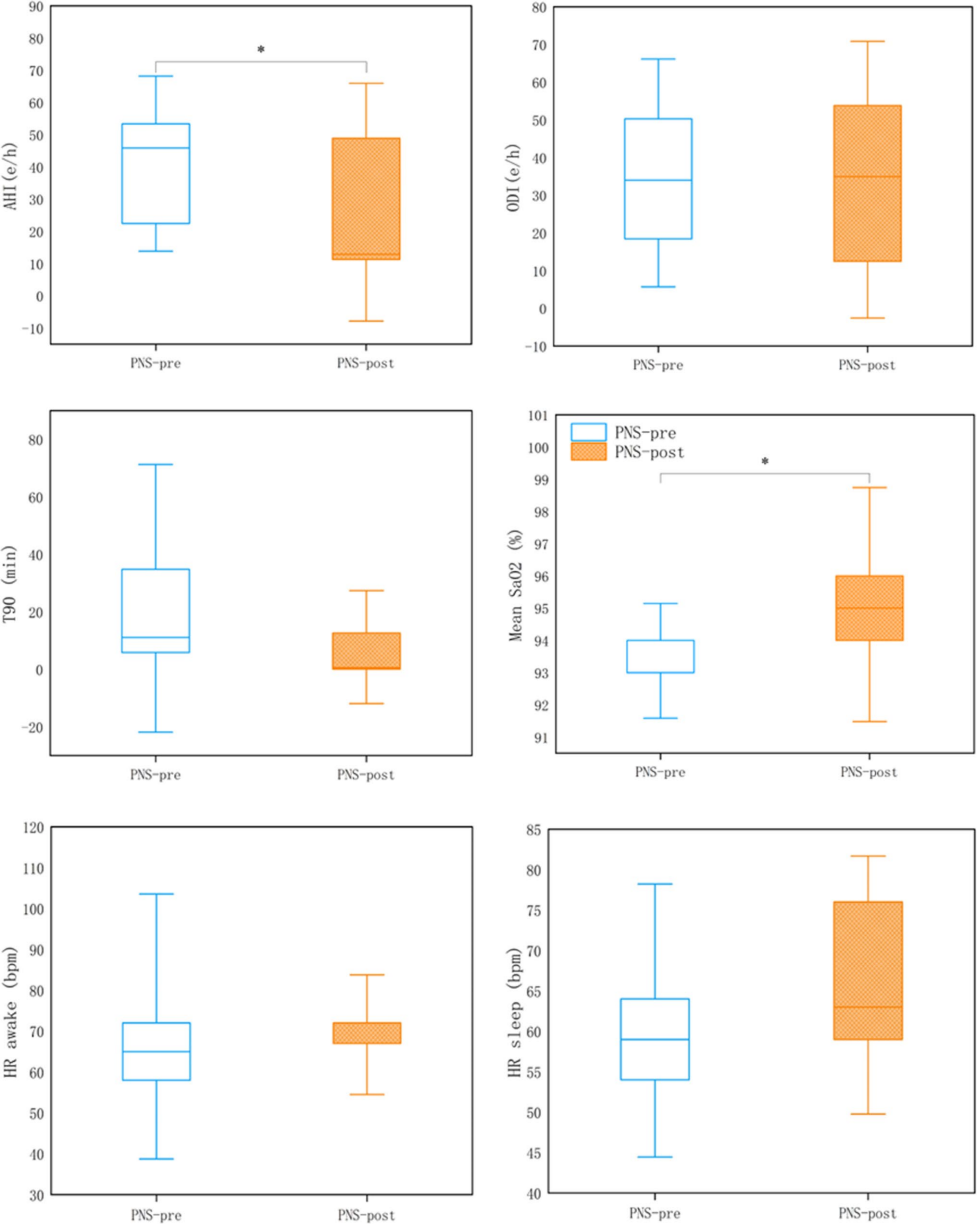
Changes of sleep parameters after PNS treatment. * *p* < 0.05**.** AHI apnea–hypopnea index, HR heart rate, ODI oxygen desaturation index, SaO2 arterial oxygen saturation, T90 time spent with oxygen saturation < 90%

The PNS led to significant improvements in the severity of CSA, including decreased AHI (*p* = 0.02) (Fig. 2 and Table 2). After PNS, mean SaO2 was increased significantly (*p* = 0.03) in these individuals (Table 2). Using established categories of disease severity based on AHI, categorical reductions were also noted in the severity of sleep apnea following unilateral PNS treatment (Table 3). During the two-night trial follow-up, no significant adverse events occurred in our research.

**Table 2:** Changes of CSA before and after PNS treatment

	Pre-PNS	Post-PNS	P-value
Overall population (n = 9)			
Total sleep time (min)	273 ± 85	298 ± 64	0.45^a^
AI (e/h)	36 ± 20	28 ± 26	0.21^b^
AHI (e/h)	41 ± 18	29 ± 25	**0.02**^**b**^
HI (e/h)	5 ± 5	6 ± 4	0.52^a^
ODI (e/h)	36 ± 20	34 ± 25	0.64^a^
Mean SaO2 (%)	93 ± 1	95 ± 2	**0.03**^**a**^
Min SaO2 (%)	85 ± 6	86 ± 9	0.55^a^
PLMI (e/h)	11 ± 10	21 ± 17	0.05^a^
T90 (min)	12 ± 13	8 ± 13	0.50^b^
REM (%)	15 ± 6	15 ± 5	0.98^a^
HR awake (bpm)	75 ± 23	69 ± 10	0.48^a^
HR sleep (bpm)	64 ± 12	66 ± 11	0.08^a^

Bold data represent *P* < 0.05

P^a^ represents the paired *t* test; P^b^ represents Wilcoxon test

*AI* apnea index, *AHI* apnea–hypopnea index, *HI* hypopnea index, *HR* heart rate, *ODI* oxygen desaturation index, *PLMI* periodic limb movement index, *REM* rapid eye movement, *T90* time spent with oxygen saturation < 90%

**Table 3:** Categorical changes of sleep apnea severity based on the AHI

Severity/AHI (e/h)	Pre-PNS (*n* = 9)	Post-PNS (*n* = 9)
Mild (< 15)	0	5 (56%)
Moderate (15–30)	3 (33%)	0
Severe (> 30)	6 (66%)	4 (44%)

*AHI* apnea–hypopnea index

## Discussion

CSA, typically associated with symptomatic HF, is widely observed in clinical practice and associated with a poor outcome. Currently, PAP is the standard treatment for CSA. Clinical trials using PAP for treating CSA have yielded contradictory outcomes [15, 16]. The CANPAP trial was a randomized, outcome study that assessed the efficacy of CPAP treatment for CSA in HF subjects; this study revealed no benefits of CPAP [17]. A post hoc examination of the trial’s data suggested that mortality could be reduced if CPAP therapy is associated with an early and considerable reduction in AHI. The mean AHI in the adaptive servo-ventilation (ASV) group at 12 months was 6.6 e/h [18]. The incidence of the primary endpoint was not substantially different between the ASV and control groups. In the ASV group, overall mortality and cardiovascular mortality were considerably greater than in the control group [19]. When compared with PAP, the benefit of PNS includes a natural breathing pattern by a diaphragmatic stimulation. As a result, physiological effects of diaphragmatic stimulation do not have similar negative hemodynamic effects on cerebral hemodynamics as PAP breathing (e.g., increased intrathoracic pressure affecting right and left ventricular preload and afterload) [20–22]. Prospective self-controlled studies such as PNS comparison can be recommended to the same patient, with a pause after PAP treatment [23]. The recent approval of the PNS system in Europe and USA provides new hope for patients with CSA. Fudim et al. used pooled individual data from the pilot (*n* = 57) and pivotal (*n* = 151) studies of the Remedē System in patients with predominant moderate to severe CSA. At 6 months, PNS reduced AHI by a median of − 22.6 e/h (25th and 75th percentiles; − 38.6 and − 8.4, respectively); PNS decreases CSA severity and sleep quality considerably. Significant and long-term reductions in key predictors of CSA severity, such as AHI, CAI, and 4% ODI, established the feasibility and therapeutic efficacy of PNS for CSA [24–26]. The degree of AHI and the reduction in AHI are related, for example, to improved outcomes in patients with obstructive or CSA. It remains to be determined whether reductions in crucial sleep parameters, symptoms, and heart function by the Remedē System can have a positive effect on cardiovascular results [18, 27]. Implant success and procedural complication rates were improved from the pilot study to the pivotal phase. Increased operator experience, improved leads, and updated implantation techniques may contribute to the rate of implant success [28].

Our study showed that PNS can be utilized to treat CSA in individuals with HF, leading to a substantial decrease in AHI. Those with the most severe sleep apnea, as defined by an AHI > 30 e/h, have the highest mortality rates [29]. In our study, the proportion of subjects with severe sleep apnea decreased from 66 to 44% when PNS was administered. During the therapeutic night, five patients (55%) had an AHI < 15 e/h. Previous research divided 151 suitable patients into treatment (*n* = 73) or control (*n* = 78) groups. Six months later, those in the treatment group had an AHI reduction from baseline that was higher than or equal to 55%, whereas those in the control group did not achieve this reduction. Significant improvement in reducing the severity of CSA, improvements in arousal indices as well as in rapid eye movement sleep, PGA scores, and ESS were observed with PNS. Consistent improvements in oxygenation and quality of life support the clinical relevance of this therapy, making PNS a potential treatment for CSA [25]. The results of the trial showed that only two patients were unable to adapt to the treatment. The therapy was well tolerated. The first implantation success rate was very high. Despite lead dislodgement, it was comparable to other implantable devices using the transvenous lead technique. A total of 138 (91%) of 151 patients experienced no serious-related side events at 12 months [25]. Our study did not detect an increased mortality in HF patients after PNS; however, past studies have shown a signal of increased mortality in CSA patients treated with PNS [19, 30]. In Dariusz’s research, only five major adverse events occurred during the 12 months of follow-up. There were no deaths as a result of serious adverse events linked to the device or procedure. None of these incidents were fatal [31]. The safety of any new medical device must be evaluated over time.

Our study had limitations. This is a single-center and non-randomized trial. We only evaluated a two-night therapy with a limited sample size, and all patients were men (a high prevalence of CSA in male patients with HF). The design of the study did not allow us to fully evaluate the potential complications of this therapy, such as its potential to interfere with pre-existing implanted cardiac devices. In addition, a few individuals were excluded due to issues with the lead placement. Notably, many patients with HF who have CSA may also have obstructive apnea; future randomized, controlled trials are needed to obtain stronger evidence.

## Conclusion

In our non-randomized study, the use of unilateral transvenous PNS may reduce the severity of CSA, providing a new approach for the treatment of CSA in patients with HF.

## Data Availability

The datasets generated and/or analyzed during the current study are available from the corresponding author on reasonable request.
